# General anaesthesia *versus* monitored anaesthesia care and postoperative outcome in patients with elevated right ventricular systolic pressure undergoing low-risk surgical procedures: a retrospective single-centre cohort study

**DOI:** 10.1016/j.bjao.2026.100529

**Published:** 2026-02-04

**Authors:** Maziar M. Nourian, Amelie Delaporte, Theodora Wingert, Nancy M. Boulos, Tristan Grogan, Emily Methangkool, Jacques Neelankavil, Louis A. Saddic, Maxime Cannesson, Olaf Mercier, Soban Umar, Marc Humbert, Laurent Savale, Alexandre Joosten

**Affiliations:** 1 Department of Anaesthesiology & Perioperative Medicine, David Geffen School of Medicine, University of California, Los Angeles, CA, USA; 2 Department of Medicine Statistics Core, David Geffen School of Medicine, University of California, Los Angeles, CA, USA; 3 Department of Thoracic Surgery and Heart-Lung Transplantation, Hôpital Marie Lannelongue, Le Plessis-Robinson, France; 4 Faculty of Medicine, Paris-Saclay University, Le Kremlin-Bicêtre, France; 5 INSERM UMR_S 999, Le Kremlin-Bicêtre, France; 6 Department of Pneumology and Respiratory Intensive Care, Bicêtre hospital, Le Kremlin-Bicêtre, France

**Keywords:** anaesthesia, general, conscious sedation, postoperative complications, pulmonary hypertension, right ventricular function, perioperative care, outcome assessment, health care

## Abstract

**Background:**

Pulmonary hypertension, often indicated before surgery by elevated right ventricular systolic pressure (RVSP) on echocardiography, is associated with increased perioperative risk. For low-risk procedures in which either general anaesthesia or monitored anaesthesia care may be selected, comparative data on postoperative outcomes are limited.

**Methods:**

We conducted a single-centre retrospective observational cohort study of 4962 adults with RVSP >50 mm Hg who underwent low-risk procedures. Eligible procedures included endoscopy, bronchoscopy, catheterisation laboratory interventions, and peripheral surgeries. The primary outcome was a composite of acute kidney injury, acute myocardial injury, or 30-day mortality. Analyses used multivariable logistic regression and inverse probability of treatment weighting based on preoperative covariates. Intraoperative variables were evaluated as potential mediators in sensitivity analyses.

**Results:**

Of the 4962 patients, 2473 received general anaesthesia and 2489 received monitored anaesthesia care. In preoperative-adjusted models, general anaesthesia was associated with higher odds of major complications (odds ratio, 1.24; 95% confidence interval [95% CI], 1.02–1.51; *P*=0.030). A doubly robust inverse probability weighted model produced similar results (odds ratio, 1.36; 95% CI, 1.08–1.70; *P*=0.0076), with estimated risks of 39.7% *vs* 32.7%. Incorporating intraoperative factors such as anaesthesia duration, vasopressor use, and fluid balance attenuated the association (odds ratio, 1.00; 95% CI, 0.81–1.23). No significant interactions were observed by emergency status or preoperative pulmonary hypertension-targeted therapy.

**Conclusions:**

After adjustment for preoperative risk and evaluating intraoperative mediators, we found no evidence of a statistically significant difference in major postoperative complications between general anaesthesia and monitored anaesthesia care in adults with elevated RVSP undergoing low-risk procedures.

Pulmonary hypertension (PH) encompasses a group of disorders marked by elevated pulmonary arterial pressure resulting from diverse pathophysiological mechanisms. PH significantly increases perioperative risk and poses substantial challenges for anaesthetic management, with higher rates of morbidity and mortality.^1^ Patients with PH have an increased risk of postoperative complications such as myocardial injury, acute kidney injury (AKI), and death.^2^^,^^3^

Currently, PH is defined as a mean pulmonary artery pressure above 20 mm Hg measured by right heart catheterisation; however, few patients presenting for an elective low-risk procedure may have a right heart catheterisation before surgery.^4^ Instead, elevated right ventricular systolic pressure (RVSP) on transthoracic echocardiography is commonly used as a surrogate marker. An RVSP >50 mm Hg suggests a high probability of PH and often triggers concern during anaesthetic planning.^5^^,^^6^

For low-risk procedures, such as gastrointestinal endoscopy, bronchoscopy, catheter-based interventions, and certain outpatient surgical cases, either general anaesthesia or monitored anaesthesia care may be appropriate. Monitored anaesthesia care refers to anaesthesia care delivered by an anaesthesia professional involving titrated sedation, anxiolysis, and physiologic monitoring with preservation of spontaneous ventilation. Evidence is lacking to inform whether one anaesthetic technique is preferable for patients with elevated RVSP undergoing such procedures. In clinical practice, this leads to variation in management and uncertainty about the safest approach.

This single-centre retrospective observational cohort study aims to evaluate whether anaesthetic technique, general anaesthesia or monitored anaesthesia care, affects major postoperative outcomes in patients with elevated RVSP undergoing low-risk surgeries. We hypothesised that there would be no significant difference in the rate of major complications, including AKI, acute myocardial injury, or 30-day mortality, between the two anaesthetic approaches.

## Methods

### Study population

This retrospective observational single-centre (Ronald Reagan UCLA Medical Center) cohort study was approved by the institutional review board at the University of California Los Angeles on 24 June 2024, under the reference IRB#24-000925, with a waiver of patient’s consent and is reported in accordance with the Strengthening the Reporting of Observational Studies in Epidemiology (STROBE) guidelines (Supplementary Table 1). All consecutive adult patients were identified from our perioperative data warehouse between April 2013 and February 2025 if they underwent low-risk procedures that could be performed with either general anaesthesia or monitored anaesthesia care, with or without regional anaesthesia, and had an RVSP >50 mm Hg. Included procedures encompassed gastrointestinal endoscopy, pulmonary bronchoscopy, catheterisation lab cases, peripheral orthopaedic or vascular procedures, select urology and ophthalmology surgeries, dentistry, plastic surgery, and otolaryngology. These procedures were specifically chosen because anaesthesiologists can reasonably choose between general anaesthesia and monitored anaesthesia care without significantly influencing surgeons’ satisfaction. Whether the patient received general anaesthesia or monitored anaesthesia care, patients had standard ASA 5 monitors and noninvasive blood pressure measurement every 3 min.

Exclusion criteria included cardiac or obstetric surgeries, patients younger than 18 yr, and procedures for which general anaesthesia is the only viable option (e.g. laparoscopic appendectomy, or cholecystectomy). Patient, clinical, diagnostic, and postoperative data were collected. Covariates used for adjustment or matching included age, sex, anaesthetic type, smoking status, ASA physical status classification, emergency procedure status (yes/no), history of chronic obstructive pulmonary disease (COPD), sleep apnoea, heart failure, coronary artery disease, diabetes, preoperative serum creatinine, and BMI. ICU admission was captured as a binary postoperative outcome; the database does not differentiate planned *vs* unplanned ICU admissions.

An RVSP cut-off of 50 mm Hg was chosen, as it has been shown to be consistent with a high probability of PH.^5^ This study was not designed to phenotype PH or to distinguish among PH Groups 1–5. Instead, our objective was to evaluate whether anaesthetic technique (general anaesthesia *vs* monitored anaesthesia care) was associated with major postoperative complications in patients presenting for low-risk surgery with an elevated RVSP, often an incidental or newly identified finding. Determining PH subtype would require invasive haemodynamics and specialised testing, which fell outside the scope of this analysis. RVSP was obtained from the most recent transthoracic echocardiogram performed before the surgical procedure; postoperative studies and historical measurements outside the perioperative period were excluded. Of note, 89% of patients had their RVSP measured within 12 months before the procedure.

### Primary outcome

The primary outcome was a composite of major postoperative complications, including AKI defined using the Kidney Disease: Improving Global Outcomes (KDIGO) criteria (without urine output criteria),^7^ acute myocardial injury, and all-cause mortality within 30 days of surgery. Acute myocardial injury was defined as either an increase in high-sensitivity troponin concentration within the first 7 postoperative days, according to the definition of ‘myocardial injury and infarction associated with noncardiac procedures’ set forth in the Fourth Universal Definition of Myocardial Infarction (2018), or a myocardial infarction within the first 7 postoperative days.^8^ These composite outcomes were selected because they represent major complications that are reliably captured and well documented in our database. Of note, high-sensitivity troponin testing at our institution was obtained routinely for this type of patient with a high risk of PH.

### Secondary outcome

The secondary outcomes included hospital length of stay, ICU admission, and 30-day hospital readmission rates.

### Bias and strategies to mitigate bias

Given the retrospective observational design, several sources of bias were considered. Confounding by indication was anticipated, as anaesthetic technique was not randomly assigned and may reflect patient, procedural, or clinician factors associated with postoperative risk. To mitigate this, we selected preoperative covariates *a priori* based on clinical relevance and previous literature and applied both multivariable regression and inverse probability of treatment weighting with doubly robust estimation. Selection bias was minimised by including all consecutive eligible patients meeting predefined inclusion criteria over the study period. Information bias was addressed through the use of standardised electronic health record-derived variables and outcome definitions. Major postoperative outcomes were selected based on their clinical relevance and reliable capture within the institutional database. To avoid inappropriate adjustment for post-treatment variables, intraoperative factors were considered potential mediators rather than confounders and were therefore excluded from primary models. These variables were instead evaluated in predefined sensitivity analyses to explore their influence on the observed associations.

### Statistical analysis

Baseline characteristics and unadjusted outcome rates were compared between general anaesthesia and monitored anaesthesia care groups using univariate statistics. Categorical variables were summarised as frequencies and percentages and compared between groups using χ^2^ tests. Continuous variables were reported as means with standard deviations or medians with interquartile ranges and compared between groups using the Student’s *t*-test. To evaluate the association between anaesthetic technique and the primary composite outcome, we first fit a preoperative covariate-adjusted logistic regression model. Covariates were selected *a priori* as potential confounders based on clinical knowledge and previous literature, reflecting factors associated with both anaesthetic technique selection and postoperative cardiopulmonary risk. These included age, sex, ASA physical status, emergency procedure status, BMI, estimated glomerular filtration rate, serum creatinine, dialysis status, procedure category, preoperative PH therapies (endothelin-receptor antagonists, prostacyclin analogues), and calendar year. Our causal framework conceptualised anaesthetic technique (general anaesthesia *vs* monitored anaesthesia care) as the exposure and major postoperative complications as the outcome. Preoperative patient and procedural characteristics were treated as confounders, whereas intraoperative variables (e.g. anaesthesia duration, invasive monitoring, vasopressor use, fluid balance) were considered post-treatment mediators on the causal pathway. Accordingly, primary models adjusted only for preoperative confounders, and intraoperative factors were evaluated separately in predefined sensitivity analyses to explore potential mediation effects. We report odds ratios (ORs) with patient-cluster-robust standard errors.

Because confounding by indication was anticipated, we next estimated inverse probability of treatment weights (IPTWs) using the same preoperative covariates (average treatment effect, stabilised weights). Covariate balance was assessed using standardised mean differences, and effective sample sizes were reported. We then fit a doubly robust weighted outcome model including the same preoperative covariates.

Intraoperative variables (e.g. anaesthesia duration, arterial line placement, vasopressor administration, fluid balance) were considered potential mediators of the effect of general anaesthesia *vs* monitored anaesthesia care, and therefore were not included in the primary models. These variables were instead incorporated into two sensitivity analyses:

(1) a preoperative model that additionally adjusted for anaesthesia time and
(2) a controlled direct effect model that adjusted for anaesthesia time, arterial line placement, any vasopressor use, and a natural spline for fluid balance.

Adjusted marginal risks and 95% confidence intervals (95% CIs) were derived via g-computation using the emmeans package. To assess heterogeneity of treatment effect, we evaluated interactions of anaesthetic technique with emergency status and preoperative PH therapy. Secondary outcomes (ICU admission, 30-day readmission) were modelled with logistic regression; hospital length of stay was modelled using median regression. There were no missing values for baseline characteristics and intraoperative variables. A multiple imputation sensitivity analysis (*m*=5) was performed for covariates; imputation models excluded anaesthesia type and outcome values. Baseline serum creatinine was available for 99.1% of patients, and postoperative creatinine values sufficient to evaluate AKI (using KDIGO creatinine criteria only) were available for 96.4%. High-sensitivity troponin measurements within 7 days after surgery were available for 81.7% of patients overall (97.5% in 2020–2025, 64.2% in 2013–2019). Thirty-day vital status was available for 100% of patients. Two-sided *P*-values <0.05 were considered statistically significant. Statistical analyses were performed using R version 4.4.3 (R Foundation for Statistical Computing, Vienna, Austria). Because this was a retrospective observational cohort study, the sample size was determined by the number of consecutive eligible patients available in the institutional database during the predefined study period. No formal *a priori* sample size or power calculation was performed. The resulting cohort size provides adequate precision to estimate associations between anaesthetic technique and major postoperative complications and to support multivariable and inverse probability-weighted analyses.

## Results

**Fig 1:**
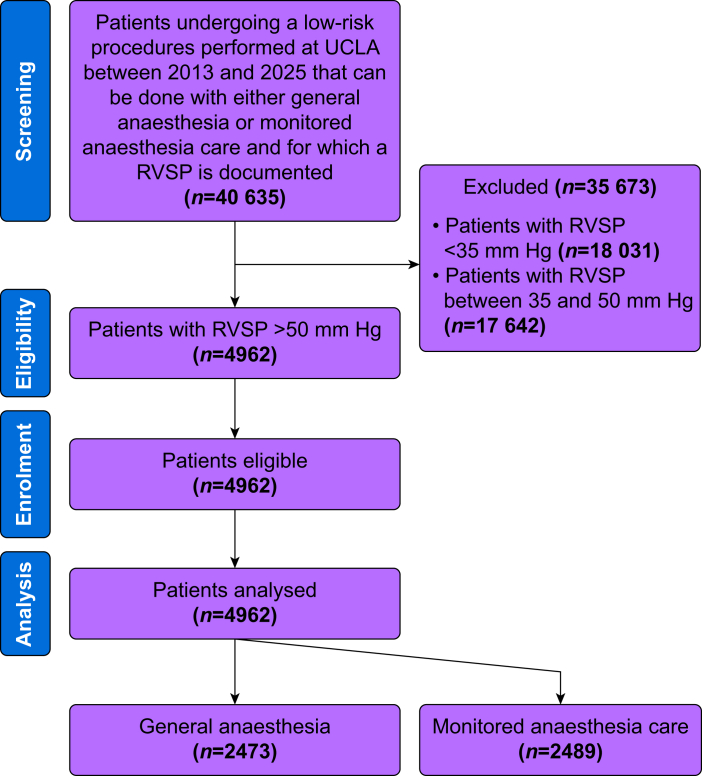
Flow chart. RVSP, right ventricular systolic pressure; UCLA, University of California, Los Angeles.

A total of 40 635 patients underwent low-risk procedures at UCLA that could be performed under either general anaesthesia or monitored anaesthesia care, and for whom a documented RVSP was available. After exclusion of 18 031 patients with RVSP <35 mm Hg and 17 642 patients with RVSP between 35 and 50 mm Hg, 4962 patients with RVSP >50 mm Hg remained and were included in the final analysis (Fig. 1). Of these, 2489 underwent monitored anaesthesia care and 2473 underwent general anaesthesia. Characteristics of patients with RVSP >50 mm Hg undergoing monitored anaesthesia care *vs* general anaesthesia are shown in Table 1. There was no statistically significant difference between monitored anaesthesia care *vs* general anaesthesia in terms of co-morbidities such as diabetes mellitus, coronary artery disease, chronic heart failure, obstructive sleep apnoea, use of preoperative dialysis, and COPD. Preoperative left ventricular ejection fraction, use of preoperative furosemide, use of endothelin-receptor antagonists, and use of prostacyclin analogues are also presented in Table 1. Importantly, the proportion of prostacyclin use was higher in the general anaesthesia group, suggesting a greater prevalence of precapillary PH (a high-risk population) in this group.

**Table 1:** Baseline characteristics. COPD, chronic obstructive pulmonary disease; GFR, glomerular filtration rate; RVSP, right ventricular systolic pressure.

Variables	Overall	Monitored anaesthesia care	General anaesthesia	*P*-value
	(*n*=4962)	(*n*=2489)	(*n*=2473)	
Age (yr)	70 (58–79)	72 (62–80)	67 (54–78)	<0.001
Weight (kg)	80 (66–82)	80 (66–82)	80 (67–82)	0.012
Height (inches)	66 (62–69)	66 (63–69)	65 (62–69)	0.012
ASA 3	3145 (63.4)	1567 (63.0)	1578 (63.8)	0.022
ASA 4	1792 (36.1)	916 (36.8)	876 (35.4)	
Emergency surgery, *n* (%)	186 (3.7)	49 (2.0)	137 (5.5)	<0.001
Female sex, *n* (%)	2458 (49.5)	1262 (50.7)	1196 (48.4)	0.099
Diabetes, *n* (%)	1664 (33.5)	831 (33.4)	833 (33.7)	0.825
Coronary artery disease, *n* (%)	1744 (35.1)	914 (36.7)	830 (33.6)	0.02
Congestive heart failure, *n* (%)	2653 (53.5)	1345 (54.0)	1308 (52.9)	0.418
Obstructive sleep apnoea, *n* (%)	1655 (33.4)	869 (34.9)	786 (31.8)	0.019
Dialysis dependent, *n* (%)	280 (5.6)	132 (5.3)	148 (6.0)	0.298
COPD, *n* (%)	104 (2.1)	49 (2.0)	55 (2.2)	0.53
Preoperative creatinine (mg dl^−1^)	1.2 (0.9–1.7)	1.2 (0.9–1.8)	1.1 (0.8–1.6)	0.022
GFR (ml min^−1^/1.73 m^2^)	60 (37–86)	58 (35–84)	63 (38–88)	<0.001
Patients GFR <60, *n* (%)	2438 (49.1)	1290 (51.8)	1148 (46.4)	<0.001
Type of surgery, *n* (%)				<0.001
Endoscopy	1234 (24.9)	762 (30.6)	472 (19.1)	
Bronchoscopy	424 (8.5)	90 (3.6)	334 (13.5)	
Cath lab	1246 (25.1)	658 (26.4)	588 (23.8)	
Orthopaedics	355 (7.2)	96 (3.9)	259 (10.5)	
Ophthalmology	417 (8.4)	400 (16.1)	17 (0.7)	
Otolaryngology	122 (2.5)	15 (0.6)	107 (4.3)	
Urology	222 (4.5)	35 (1.4)	187 (7.6)	
Plastic surgery	28 (0.6)	7 (0.3)	21 (0.8)	
Gynaecology	62 (1.2)	22 (0.9)	40 (1.6)	
Dentistry	330 (6.7)	73 (2.9)	257 (10.4)	
Vascular surgery	413 (8.3)	283 (11.4)	130 (5.3)	
Surgical oncology	68 (1.4)	26 (1.0)	42 (1.7)	
Other	41 (0.8)	22 (0.9)	19 (0.8)	
RVSP (mm Hg)	57 (52–67)	57.0 (52–66)	57 (52–68)	0.075
Left ventricular ejection fraction (%)	55 (50–60)	55 (50–60)	55 (55–60)	>0.999
Treatment, *n* (%)				
Furosemide	3769 (76.0)	1832 (73.6)	1937 (78.3)	<0.001
Endothelin-receptor antagonist	340 (6.9)	173 (7.0)	167 (6.8)	0.783
Prostacyclin	684 (13.8)	256 (10.3)	428 (17.3)	<0.001

Intraoperative data are depicted in Table 2. The median (Q1–Q3) anaesthesia time was 72 (44–126) min for monitored anaesthesia care *vs* 145 (78–191) min for general anaesthesia (*P*<0.001). The duration of intraoperative hypotension (defined as a mean arterial pressure <60 mm Hg) did not differ between both strategies (*P*=0.403).

**Table 2:** Perioperative variables.

Variables	Overall	Monitored anaesthesia care	General anaesthesia	*P*-value
	(*n*=4962)	(*n*=2489)	(*n*=2473)	
Anaesthesia time (min)	101 (55–170)	72 (44–126)	145 (78–191)	<0.001
Mean arterial pressure <60 (min)	0 (0–6)	0 (0–6)	0 (0–6)	0.403
Hospital length of stay (h)	27 (5–151)	8 (4.0–116)	42 (8–187)	<0.001
ICU admission, *n* (%)	290 (5.8)	135 (5.4)	155 (6.3)	0.205
Hospital readmission within 30 days, *n* (%)	1176 (23.7)	626 (25.2)	550 (22.2)	0.016
Composite complications (unadjusted) , *n* (%)	653 (13.2)	262 (10.5)	391 (15.8)	<0.001
Acute kidney injury, *n* (%)	351 (7.1)	137 (5.5)	214 (8.7)	<0.001
Myocardial injury, *n* (%)	305 (6.1)	127 (5.1)	178 (7.2)	0.002
Mortality, *n* (%)	164 (3.3)	47 (1.9)	117 (4.7)	<0.001

The overall unadjusted composite of major postoperative complications was 10.5% for monitored anaesthesia care *vs* 15.8% for general anaesthesia (*P*<0.001) and can be found in Table 2. In the preoperative only model, general anaesthesia was associated with higher odds of complications (OR, 1.24; 95% CI, 1.02–1.51; *P*=0.030). The IPTW doubly robust model similarly showed higher odds with general anaesthesia (OR, 1.36; 95% CI, 1.08–1.70; *P*=0.0076), with good covariate balance after weighting (all absolute standardised mean differences ≲0.03) and effective sample sizes of 1757.9 (monitored anaesthesia care) and 1158.9 (general anaesthesia). Model-based marginal risks in the IPTW doubly robust analysis were 32.7% for monitored anaesthesia care *vs* 39.7% for general anaesthesia.

Adding anaesthesia time to the preoperative model attenuated the general anaesthesia association (OR, 1.00; 95% CI, 0.81–1.23; *P*=0.991). Similarly, the controlled direct-effect model, including arterial line placement, any vasopressor use, and fluid-balance spline, yielded an OR of 1.00 (95% CI, 0.81–1.23; *P*=0.997). Interactions of general anaesthesia with emergency status (*P*=0.38) and with preoperative PH therapy (*P*=0.67) were non-significant (Table 3).

**Table 3:** Sensitivity analyses for general anaesthesia *vs* monitored anaesthesia care and the primary composite outcome. 95% CI, 95% confidence interval.

Model	OR (95% CI)	*P-*value
Preoperative covariates only	1.24 (1.02–1.51)	0.030
Anaesthesia time	1.00 (0.81–1.23)	0.991
Controlled Direct Effect (CDE)	1.00 (0.81–1.23)	0.997
Inverse probability of treatment weighting doubly robust	1.36 (1.08–1.70)	0.0076

When analysing secondary outcomes, general anaesthesia was not associated with ICU admission (OR, 0.95; 95% CI, 0.73–1.24; *P*=0.709). General anaesthesia was associated with lower 30-day readmission (OR, 0.86; 95% CI, 0.74–1.00; *P*=0.045). In median regression, general anaesthesia was associated with +4.21 h longer hospital stay (*P*<0.001). Multiple imputation sensitivity analyses produced results consistent with complete-case and weighted analyses.

## Discussion

In this single-centre cohort of 4962 adults with elevated RVSP (>50 mm Hg) undergoing low-risk procedures in which either anaesthetic technique was feasible, both conventional multivariable adjustment and inverse probability weighted doubly robust modelling indicated higher complication risk under general anaesthesia relative to monitored anaesthesia care. The general anaesthesia association, however, attenuated to null after adjustment for intraoperative variables, consistent with mediation through peri-procedural pathways (e.g. longer anaesthesia duration, invasive monitoring, and vasoactive support), over-adjustment, or both when conditioning on factors downstream of treatment. We found no evidence of heterogeneity by emergency status or preoperative PH therapy. Secondary endpoints were mixed: general anaesthesia was associated with lower 30-day readmission but longer length of stay, and no difference in ICU admission was observed. Because planned and unplanned ICU admissions could not be distinguished, ICU disposition should be interpreted cautiously, as protocolised monitoring practices or service-level pathways may influence admission patterns independent of anaesthetic technique.

These results suggest that the apparent increased risk associated with general anaesthesia in preoperative confounder-adjusted models may be partially explained by intraoperative processes rather than anaesthetic technique alone. Thus, preoperative case mix and perioperative management appear to jointly drive postoperative risk, underscoring the importance of patient selection, procedure duration, monitoring strategy, and haemodynamic management rather than a uniform preference for one anaesthetic modality.

Patients with elevated RVSP are known to be at increased risk of perioperative cardiopulmonary complications, including acute myocardial injury, right heart failure, AKI, and death.^1^^,^^9^, ^10^, ^11^, ^12^ Elevated pulmonary vascular pressures impose additional right ventricular afterload, reducing forward flow and rendering patients vulnerable to hypotension, hypoxia, and hypercarbia, physiologic disturbances that may arise under either general anaesthesia or monitored anaesthesia care. Previous reports demonstrate that each 5-mm Hg increase in RVSP confers incremental mortality risk.^13^ Our findings are consistent with this observation: elevated RVSP itself appears to be an important risk marker in both general anaesthesia- and monitored anaesthesia care-treated patients, reinforcing its prognostic relevance.^14^

Previous literature on anaesthetic technique in PH is limited by small samples, higher-risk procedures, heterogeneous endpoints, or insufficient adjustment for confounding.^15^^,^^16^ Our study adds to this literature by examining nearly 5000 patients across procedures where anaesthetic choice is discretionary. The preoperative only model and IPTW doubly robust model demonstrated modestly increased risk under general anaesthesia, but this difference disappeared after incorporating intraoperative characteristics. This trajectory highlights the importance of distinguishing pre-treatment confounders, which should be adjusted for, from post-treatment mediators, which lie on the causal pathway and require careful interpretation. The attenuation toward the null after mediator adjustment should not be interpreted as evidence that general anaesthesia is ‘equally safe’, but rather that perioperative processes likely account for some of the observed excess risk under general anaesthesia. This distinction was central to reviewer concerns and is specifically addressed in our analytic framework.

The physiological trade-offs between monitored anaesthesia care and general anaesthesia are well recognised. General anaesthesia secures the airway and may permit more controlled ventilation and anaesthetic depth but may also increase pulmonary vascular resistance via positive pressure ventilation, volatile agents, and higher opioid doses. Monitored anaesthesia care preserves spontaneous ventilation and avoids airway instrumentation but may be limited by procedure duration and carries risks of hypoventilation, hypercarbia, and hypoxia in PH patients. In our cohort, general anaesthesia cases were associated with longer anaesthesia times, which may reflect procedural complexity or institutional practice rather than intrinsic anaesthetic risk. Additionally, rates of intraoperative hypotension were similar between groups, suggesting no clear haemodynamic advantage of either modality in this context. Although invasive arterial monitoring may have differed between groups, our primary hypotension metric (minutes with MAP <60 mm Hg) was derived from the full set of noninvasive and invasive recordings at the standard timestamp resolution, which reduces differential misclassification based on monitoring modality. Our results do not indicate that elevated RVSP is benign nor that it lacks prognostic value. Rather, within the limitations of this retrospective dataset, we found no statistically significant adjusted association between anaesthetic technique (general anaesthesia vs monitored anaesthesia care) and major postoperative complications in patients presenting for low-risk procedures with an elevated RVSP. These findings should not be interpreted as evidence that RVSP >50 mm Hg predicts comparable risk across PH phenotypes or surgical settings.

The external validity of our study findings should be considered in light of its single-centre design. Institutional practice patterns, including case selection, anaesthetic decision-making, perioperative monitoring, and postoperative care pathways, may differ across centres and healthcare systems. Our cohort reflects a large, heterogeneous population undergoing low-risk procedures in which anaesthetic technique is discretionary, suggesting that the findings may be applicable to similar tertiary care settings with comparable expertise and perioperative infrastructure. However, generalisability may be limited in institutions with different patient case mix, procedural complexity, or thresholds for selecting general anaesthesia *vs* monitored anaesthesia care. In particular, centres with limited experience managing patients with elevated RVSP or PH, or those caring for higher-risk surgical populations, may observe different risk profiles. Accordingly, our findings should be interpreted as informing anaesthetic decision-making in comparable low-risk procedural contexts rather than as definitive guidance across all surgical settings.

Strengths of this study include its large sample, detailed perioperative data, and robust analytic framework incorporating preoperative risk adjustment, IPTW with doubly robust estimation, mediation-aware sensitivity analyses, and interaction testing. The analytic approach allowed us to isolate preoperative confounding, evaluate potential mediators, and quantify causal contrasts relevant to clinical decision-making.

Several limitations warrant discussion. As a retrospective single-centre study, residual confounding is possible, and results may not generalise to other institutions. Additionally, as with most retrospective observational studies, sample size was not based on a predefined target effect size but rather reflects the available population meeting eligibility criteria. RVSP was estimated by transthoracic echocardiography; although clinically important and noninvasive, it is less precise than right heart catheterisation and insufficient for definitive PH phenotyping. Additionally, RVSP does not perfectly reflect haemodynamics at the time of the surgery and can introduce non-differential misclassification. Importantly, an elevated RVSP should still prompt consideration of referral to a PH centre for formal evaluation, as patients with precapillary PH (Groups 1 and 4) remain at high risk and may experience instability even during minimal sedation. Our study was not designed to phenotype PH or evaluate safety in these high-risk subgroups. We used preoperative PH-targeted therapy as a pragmatic proxy for precapillary PH; although reasonable, this approach is imperfect. Finally, intraoperative haemodynamic management practices vary and may influence outcomes.

### Conclusions

After adjusting for preoperative risk and accounting for intraoperative mediators—and recognising the limitations of observational data and residual confounding—we found no evidence of a statistically significant difference in major postoperative complications between general anaesthesia and monitored anaesthesia care in adults with elevated RVSP undergoing low-risk procedures. These findings suggest that elevated RVSP alone should not determine anaesthetic choice, and individualised decision-making remains essential. Prospective studies are needed to further clarify causal mechanisms and to guide anaesthetic best practices in PH patients across a broader spectrum of procedural risk.

## Authors’ contributions

Study design/conception: all authors

Data analysis: AJ, MMN, TG

Writing paper: MMN, AJ

Editing paper: all authors

## Funding

Department sources; National Institute of General Medical Sciences (to NMB, T32 PI Eghbali/Cannesson).

## Declarations of interest

MH reports grants from Acceleron, AOP Orphan, Janssen, Merck, and Shou Ti; consulting fees from Acceleron, Aerovate, Altavant, AOP Orphan, Bayer, Chiesi, Ferrer, Janssen, Merck, MorphogenIX, Shou Ti, and United Therapeutics; payment or honoraria for lectures, presentations, manuscript writing, or educational events from Janssen and Merck; and participation on a data safety monitoring board or advisory board with Acceleron, Altavant, Janssen, Merck, and United Therapeutics. LS is supported by a Contrat d’Interface from Assistance Publique-Hôpitaux de Paris (AP-HP)-INSERM. MC and AJ are consultants for Edwards Lifesciences, Irvine, CA, USA. The other authors declare that they have no conflicts of interest.
